# Editorial: Interstitial Lung Disease in the Context of Systemic Disease: Pathophysiology, Treatment and Outcomes

**DOI:** 10.3389/fmed.2020.644075

**Published:** 2021-01-20

**Authors:** Peter Korsten, Maximilian F. Konig, Björn Tampe, Mehdi Mirsaeidi

**Affiliations:** ^1^Department of Nephrology and Rheumatology, University Medical Center Göttingen, Göttingen, Germany; ^2^Division of Rheumatology, Department of Medicine, The Johns Hopkins University School of Medicine, Baltimore, MD, United States; ^3^Division of Pulmonary, Critical Care, and Sleep Medicine, University of Miami Miller School of Medicine, Miami, FL, United States

**Keywords:** interstitial lung disease, autoimmune diseases, rheumatoid arthritis, systemic sclerosis (scleroderma), myositis

## Introduction

Interstitial lung disease (ILD) is an umbrella term for many different disease entities causing inflammation and fibrosis of the lung parenchyma. These can be broadly divided into five categories based on etiology (1): (1) ILD related to a distinct primary disease (e.g., sarcoidosis), (2) ILD related to environmental factors (e. g., hypersensitivity pneumonitis), (3) ILD induced by drugs or irradiation, (4) idiopathic interstitial pneumonias (e.g., idiopathic pulmonary fibrosis), and (5) ILD related to connective tissue diseases (CTD) (1). While all these entities require thorough and multidisciplinary assessment to ascertain a diagnosis, establish the need for diagnostic procedures, and recommend a patient-specific treatment plan, ILDs associated with systemic diseases are particularly challenging. In many cases, optimal treatment for involvement of other organ systems needs to be balanced with the choice of ILD-directed therapies.

For the highly heterogeneous group of patients who cannot be given a definite diagnosis of an autoimmune rheumatic disease but who demonstrate certain clinical, radiographic, and/or serological features suggestive of a CTD, the term interstitial pneumonia with autoimmune features (IPAF) has been coined (2), but its clinical value remains to be defined. Due to its complexity, management of ILDs associated with systemic disease requires multidisciplinary care, including pulmonologists, rheumatologists, and radiologists, often with critical input from other specialties, such as pathologists, dermatologists, or neurologists. In this Research Topic, we hope to present novel research and state-of-the-art reviews as relevant to the care of patients with ILD and systemic diseases.

## Frequently Encountered ILDs Associated with Systemic Diseases

**Figure 1:**
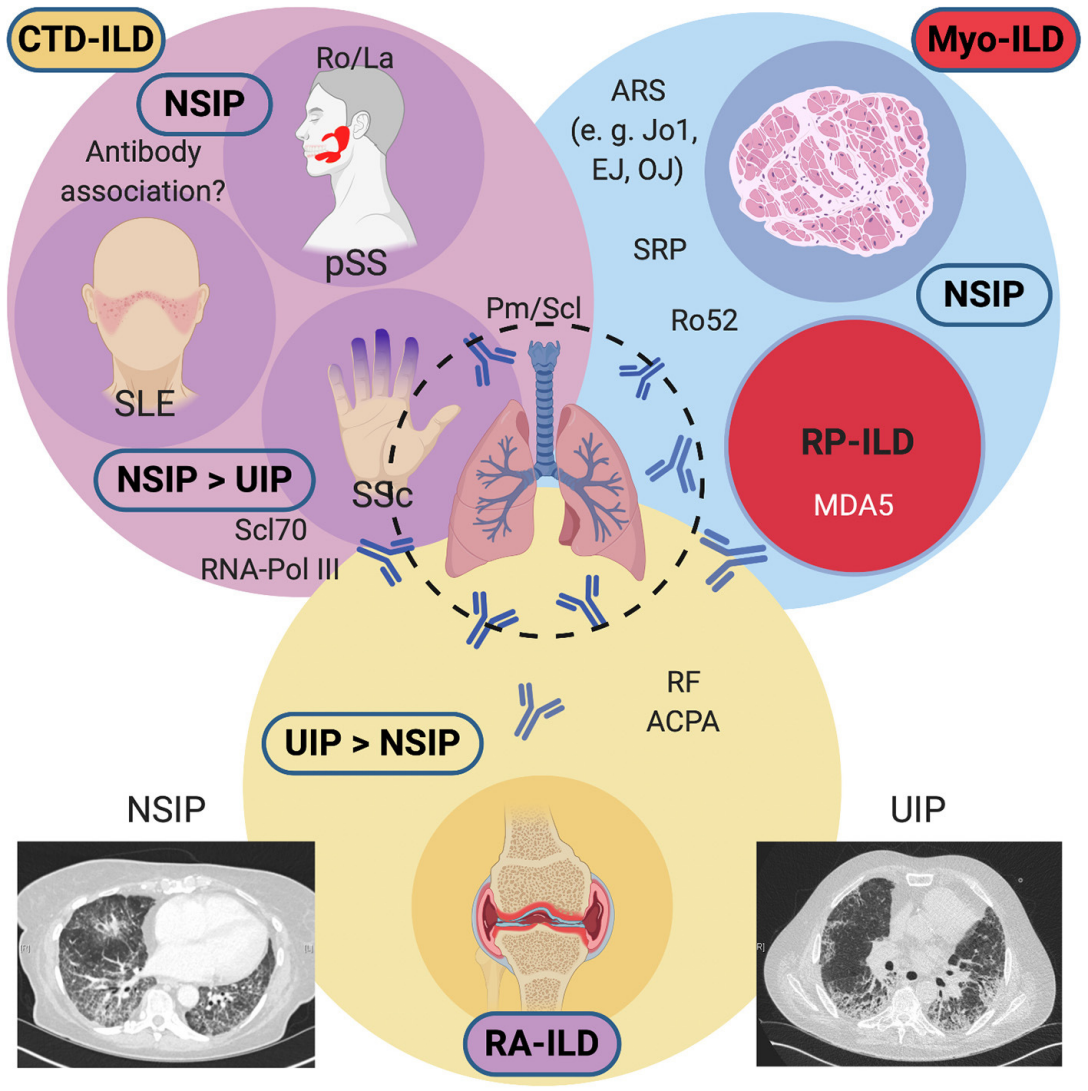
Overview of systemic diseases causing interstitial lung diseases (ILDs) and their associated antibodies. Conditions associated with a predominant NSIP pattern on HRCTs include Myositis-associated ILD (antisynthetase syndrome, anti-RO52 positive overlap myositis, or RP-ILD), Systemic Sclerosis, primary Sjögren's syndrome, and rarely, Systemic Lupus Erythematosus. In SSc, a UIP pattern may also be commonly found. RA-ILD frequently shows a UIP pattern and confers a guarded prognosis. An NSIP pattern may also be encountered on HRCTs. ACPA, anti-citrullinated peptide antibodies; ARS, aminoacyl-tRNA synthetase antibodies; CTD, connective tissue disease; HRCT, high-resolution computed tomography; MDA5; melanocyte-differentiation antigen 5; Myo, myositis; NSIP, non-specific interstitial pneumonia; Pm/Scl, polymyositis-scleroderma; pSS; primary Sjögren's syndrome; RA, rheumatoid arthritis; RF, rheumatoid factor; RNA Pol III, RNA polymerase III; RP, rapidly progressive; Scl70, topoisomerase I; SLE, systemic lupus erythematosus, Sm, Smith; SRP, signal recognition particle; SSc, systemic sclerosis; U1-snRNP, U1 small nuclear ribonucleoprotein; UIP, usual interstitial pneumonia. Created with BioRender.com.

The most frequently encountered ILDs in the setting of systemic diseases include parenchymal lung involvement with myositis (Myo-ILD), systemic sclerosis (SSc-ILD), and rheumatoid arthritis (RA-ILD). Less frequently reported are ILDs secondary to primary Sjögren's syndrome (pSS) or systemic lupus erythematosus (SLE). Figure 1 gives an overview of systemic diseases and frequently encountered autoantibodies that have been associated with the development of ILD in these diseases. For practical reasons, we find it helpful to distinguish these based on their predominant pattern on high-resolution computed tomography (HRCT), most frequently usual interstitial pneumonia (UIP) and non-specific interstitial pneumonia (NSIP). UIP changes tend to be less reversible and tend to confer a worse prognosis, whereas changes in NSIP (particularly in patients with non-fibrotic NSIP) may be more responsive to treatment. In different diseases, these lung disease patterns occur with varying frequency. In RA, UIP is often more frequently encountered than NSIP, although both may occur. Anti-cyclic citrullinated peptide (CCP) antibodies and rheumatoid factor (RF), present in 70–80% of patients with RA, are associated with RA-ILD. The same applies to SSc, where NSIP is common, but UIP also occurs. In pSS, pulmonary manifestations are relatively rare but have been reported to occur in about 16% of patients, and a consensus guideline on the diagnostic and therapeutic approach has recently been published (3). In pSS, the occurrence of a lymphocytic interstitial pneumonitis (LIP) is rare, but this radiologic pattern should prompt a search for other manifestations of pSS if ILD is the first presenting manifestation (4). ILD is common in myositis, especially in patients with antibodies directed against different aminoacyl-tRNA synthetases, a group collectively called the antisynthetase syndrome (ASyS), but also those with anti-Ro52 antibodies (5, 6). Anti-MDA5 antibodies are associated with clinically amyopathic dermatomyositis (CADM) which can present as a rapidly progressive ILD with high mortality despite aggressive immunosuppressive therapy (7). The following summarizes the papers publishes within this Research Topic.

## A Multidisciplinary Approach is Useful for the Diagnosis and Management

In the first paper, Furini et al. systematically investigated the evidence for multidisciplinary conferences (MDC) to assess ILDs. They found that most MDC evaluated patients with history taking and clinical assessment, HRCT, pulmonary function tests (PFTs), lung biopsy (in most MDCs), and serological testing. Less consensus existed on the use of additional tests, such as nailfold video capillaroscopy (NVC) and 6-min walking distance. Only seven studies evaluated the rheumatologist's role in MDCs, but the authors suggest that MDCs include serological testing and rheumatology expertise to help classify CTD-ILD and IPAF with more certainty. The second paper by Tirelli et al. reported results from a retrospective cohort study from Pavia, Italy. In their cohort, the authors found that 15% of all patients were diagnosed with CTD-ILD, 33% were classified as IPAF; the remainder had no underlying systemic disease. They found the application of a standardized screening questionnaire, laboratory testing, and the inclusion of NVC useful for detecting these entities. Karampitsakos et al. summarized the current use and ongoing clinical trials of biological therapies in sarcoidosis and idiopathic pulmonary fibrosis, which emphasizes a potentially useful role of rheumatologists in the management of ILDs given their extensive expertise in the use of these drugs and management of complications.

## Connective Tissue Disease-Associated ILD

One paper specifically reported findings in SLE, pSS, and SSc patients. Patients with SLE and pSS tend to have less ILD. Therefore, it is even more critical to identify individuals at risk and potential overlap syndromes among patients with these relatively common autoimmune rheumatic diseases. Amarnani et al. reviewed the pulmonary manifestations of SLE, including ILDs. Interestingly, the development of an ILD in SLE has not been associated with anti-dsDNA antibodies but rather anti-Ro or anti-U1-snRNP antibodies, which are also associated with mixed connective tissue disease (MCTD). It is, therefore, unclear whether these patients instead represent an overlap population. Sogkas et al. reported their single-center experience from a large cohort of pSS patients with ILD. At their center, 13% of patients were eventually diagnosed with ILD. Of note, almost two thirds (61%) were diagnosed with ILD at presentation and, surprisingly, UIP was the most commonly encountered pattern on HRCT (43%). Lastly, Mirsaeidi et al. reviewed the current treatment options for SSc-ILD, an emerging and rapidly changing topic with the recently approved anti-fibrotic nintedanib, and several ongoing clinical trials.

## Myositis-Associated ILD

The majority of papers addressed Myo-ILD. The review by Hervier and Uzunhan represents a timely and current overview of the diagnostic and therapeutic approach to Myo-ILD. This is expanded by original data from Asian cohorts on rapidly-progressive ILD (Li et al.), acute exacerbations of ILD in Myo-ILD (Liang et al.), and the understudied role of plasma exchange in the treatment of refractory Myo-ILD (Ning et al.). Finally, Korsten et al. report their findings on the efficacy of immunosuppression in ASyS-ILD. They specifically report equal usefulness of rituximab (RTX) compared to other conventional immunosuppressive drugs, especially in patients with more frequent clinical myositis and progressive or relapsing pulmonary involvement.

## Rheumatoid Arthritis-Associated ILD

The recent observation of *MUC5B* promoter variants as a risk factor for RA-ILD, similar to patients with idiopathic pulmonary fibrosis, has generated significant interest in understanding the potential role of antifibrotic strategies in the treatment of this subset of ILD (8). Emerging data from cohort studies examining different immunosuppressive drugs [e.g., abatacept or RTX; (9, 10)] is of similar interest. Fragoulis et al. provided an overview of the difficult topic of RA-ILD, which is an area of active investigation. The authors summarize data on methotrexate-induced pneumonitis and pulmonary fibrosis associated with RA.

## Author Contributions

PK wrote the first draft and created the figure. BT, MK, and MM edited and reviewed the draft. All authors approved the final version of the manuscript.

## Conflict of Interest

The authors declare that the research was conducted in the absence of any commercial or financial relationships that could be construed as a potential conflict of interest.
